# In vivo T_1_ and T_2_ relaxation time maps of brain tissue, skeletal muscle, and lipid measured in healthy volunteers at 50 mT

**DOI:** 10.1002/mrm.29009

**Published:** 2021-09-14

**Authors:** Thomas O’Reilly, Andrew G. Webb

**Affiliations:** ^1^ Department of Radiology Leiden University Medical Center Leiden the Netherlands

**Keywords:** gray matter, lipid, low‐field MRI, muscle, relaxation times, white matter

## Abstract

**Purpose:**

Low‐field (B_0_ < 0.1 T) MRI has generated much interest as a means of increased accessibility via reduced cost and improved portability compared to conventional clinical systems (B_0_ ≥ 1.5 Tesla). Here we measure MR relaxation times at 50 mT and compare results with commonly used models based on both in vivo and ex vivo measurements.

**Methods:**

Using 3D turbo spin echo readouts, T_1_ and T_2_ maps of the human brain and lower leg were acquired on a custom‐built 50 mT MRI scanner using inversion‐recovery and multi‐echo–based sequences, respectively. Image segmentation was performed based on a histogram analysis of the relaxation times.

**Results:**

The average T_1_ times of gray matter, white matter, and cerebrospinal fluid (CSF) were 327 ± 10 ms, 275 ± 5 ms, and 3695 ± 287 ms, respectively. Corresponding values of T_2_ were 102 ± 6 ms, 102 ± 6 ms, and 1584 ± 124 ms. T_1_ times in the calf muscle were measured to be 171 ± 11 ms and were 130 ± 5 ms in subcutaneous and bone marrow lipid. Corresponding T_2_ times were 39 ± 2 ms in muscle and 90 ± 13 ms in lipid.

**Conclusions:**

For tissues except for CSF, the measured T_1_ times are much shorter than reported at higher fields and generally lie within the range of different models in the literature. As expected, T_2_ times are similar to those seen at typical clinical field strengths. Analysis of the relaxation maps indicates that segmentation of white and gray matter based purely on T_1_ or T_2_ will be quite challenging at low field given the relatively small difference in relaxation times.

## 1 INTRODUCTION

There is growing interest in low‐field MRI systems as a reduced cost and lower footprint alternative/addition to clinical 1.5 Tesla (T) and 3T systems, particularly as a way of bringing MRI to low resource settings where conventional MR systems are not accessible.^1^, ^2^, ^3^ Various system designs have been proposed: many are based on Halbach and other permanent magnet array systems, either with a homogenous B_0_ field^4^, ^5^ or with a built‐in spatial encoding gradient,^6^, ^7^, ^8^, ^9^ yoked permanent magnets,^10^, ^11^, ^12^ fast field‐cycling systems,^13^ and electromagnets.^14^, ^15^ Of these systems, a number operate at magnetic field strengths between ~50 and ~80 mT. At low field, there are major advantages in terms of increased implant safety and reduced implant‐induced susceptibility artefacts,^16^ as well as reduced specific absorption rate (SAR).^17^ The major disadvantage of operating at these field strengths is the significantly reduced signal to noise ratio (SNR) due its power law dependence on the B_0_ field strength, with a coefficient of ~7/4 at low field where coil noise rather than body noise is the dominant loss‐term.^18^

Some of this loss in SNR may, however, be recovered because of favorable relaxation times and fewer limitations on RF power due to the lower specific absorption rate.^19^ In vivo T_1_ relaxation times, dominated by dipole–dipole interactions, are well known to be shorter at lower magnetic fields.^20^, ^21^, ^22^, ^23^, ^24^, ^25^ The T_1_ value is related to components of the spectral density at the Larmor frequency and twice this frequency. The general relaxation model has 2 components—free and bound water—undergoing exchange that is rapid compared to the MR measurement time scale. The fast exchange 2‐state model shows that the measured relaxation rate is the weighted average of bound and free water. As discussed in Korb and Bryant,^21^ the magnetic field dependence arises from the magnetic coupling of water protons to the solid components of the tissue, that is, water molecule exchange between specific binding sites, and to a much lesser degree proton exchange with specific groups (amines, amides, and alcohols) on protein molecules. In lipid tissue, hydrogen nuclei are present mainly in long chain triglycerides, which have relatively slow rotational and molecular motion and therefore generally have a shorter T_1_ value than tissue water.

T_2_ times show a much weaker field strength dependence,^22^ although tissues with substantial iron concentrations have significantly longer T_2_ values at lower fields.^24^, ^26^ The weak dependence is due to the fact that the frequency‐independent static component (0 order) of the spectral density functions for most tissues dominate the first‐ and second‐order terms (which are frequency‐dependent): the greater the degree to which T_2_ < T_1_, the lower the frequency dependence.

Whereas in vivo relaxation times have extensively been studied in both healthy subjects and in many pathologies at clinical field strengths, the available literature at low field strength is much sparser. Bottomley^22^ collected a large set of relaxation times acquired at different field strengths, primarily from ex vivo samples, including animal tissue. An empirical model for the relaxation time as a function of magnetic field strength was generated from this data and can be used to estimate the T_1_ relaxation times for various tissues as a function of magnetic field strength, albeit with quite a large standard deviation in the predicted values. Rooney et al^20^ performed an in vivo study on brain tissue at several different field strengths and derived a similar empirical formula for T_1_ relaxation, albeit with different coefficients than those of Bottomley. Fischer et al^23^ extended these models to take into account the actual upper and lower bounds of the relaxation time values at very low and very high fields.

In this work, we acquire in vivo T_1_ and T_2_ relaxation time maps of the brain and lower leg in healthy subjects on a custom‐built 50 mT permanent magnet‐based MRI scanner. We compare the measured values with those predicted by the models mentioned in the previous paragraph, and also with selected measurements at similar fields. We also discuss the implications for image segmentation at low field.

## 2 METHODS

### 2.1 Hardware

All data were acquired on a custom‐built 50 mT (2.15 MHz) Halbach‐based MRI scanner that was previously described in detail.^4^, ^5^ The magnet is constructed using 2948 12‐mm cuboid N48 neodymium iron boron magnets arranged in a cylindrical Halbach configuration. The magnet is 50.6 cm long and has a 27‐cm diameter bore. The magnetic field homogeneity was optimized over a 20‐cm diameter spherical volume placed at the center of the magnet. A set of 3 linear gradient coils was constructed using an target field method initially proposed by Turner,^27^ adapted for the transverse B_0_ orientation intrinsic to cylindrical Halbach arrays.^28^ A Magritek Kea2 spectrometer (Aachen, Germany) was used to generate RF and gradient waveforms and digitize the generated signals. A custom‐built 1 kW RF amplifier with 56 dB gain was used to amplify the RF pulses.^4^ The gradient waveforms were amplified using a custom‐built 3‐axis current‐controlled gradient amplifier, powered using 2 Delta Elektronika SM 18‐50 DC power supplies (Zierikzee, the Netherlands). The entire setup is placed inside a Faraday cage constructed from aluminium extrusion and 2‐mm thick aluminium plates. An RF shield is placed inside the inner surface of the bore. During in vivo experiments, the body extends out of the Faraday and couples significant amounts of electromagnetic interference into the RF coil. In order to reduce electromagnetic interference, this the body is placed under a conductive cloth (4711 series, Holland Shielding Systems BV, Dordrecht, the Netherlands).

Brain imaging was performed using an elliptical 20‐cm wide, 25‐cm tall, 15‐cm deep spiral‐solenoid head coil^15^ constructed using 0.8 mm copper wire and with a single capacitive segmentation. The bandwidth of the coil was 35 kHz when loaded with a head. Data on the lower leg were acquired using a 15‐turn, 15‐cm long, 15‐cm diameter solenoid constructed using 0.8‐mm diameter copper wire. The bandwidth of the coil was 23 kHz when the coil was loaded with the lower leg. Power optimization for both coils was performed by acquiring 16 spectra with 1 dB increments in the transmitted RF power. The area under the measured signal in the frequency domain was integrated from −1 to 1 kHz around the central frequency for each of the 16 steps, and a sinusoidal curve was fitted to the linearized power. First‐order B_0_ shimming was performed using the standard automatic shimming algorithm included with the spectrometer, which maximizes the peak of the spectrum of a nonselective FID by applying pseudo‐random gradient offsets to 3 linear gradient coils. Line widths (measured as the full width at half maximum) were less than 1 kHz on all volunteers after shimming. The center frequency of the sample was measured prior to every scan.

### 2.2 Relaxation mapping

Human studies were conducted with the approval of the institutional review board. Conventional inversion‐recovery and multiple spin‐echo sequences were used to map T_1_ and T_2_, respectively. Total imaging times were relatively long and could potentially be shortened considerably by use of more efficient sequences such as Look‐Locker,^29^ MR fingerprinting,^30^ or compressed sensing.^31^ In this preliminary work, however, the choice was made to use conventional sequences for maximum confidence in the reported values. Separate sessions were used for CSF measurements because the long T_1_ time in particular results in a long imaging session. Our imaging protocol is limited to 1 h maximum; thus, separate measurement sessions were required for each relaxation time.

Data were corrected for any frequency drift induced by heating of the magnet during the acquisition by applying appropriate phase shifts of the k‐space data. The center frequency was measured immediately before and after the scan, and the drift is assumed to be spatially homogeneous over the imaging region and linear over time. Values were typically 500 Hz over the ~45 min imaging time. All data were acquired on healthy volunteers aged 27 to 62 years (6 male, 4 female). Informed consent was obtained from all subjects prior to scanning.

### 2.3 Brain relaxation time mapping

T_1_ maps of the gray matter (GM) and white matter (WM) were reconstructed from 6 whole‐brain 3D inversion recovery scans with a turbo spin echo readout acquired with the following scan parameters: resolution: 2.5 × 2.5 × 5 mm^3^; TR/TE/TE_eff_: 1250 ms/13 ms/13 ms (center‐out Cartesian k‐space trajectory); no signal averaging; echo train length: 6; and acquisition bandwidth: 20 kHz. Scans were acquired with inversion times of 50, 100, 150, 200, 300, and 500 ms; total scan duration was around 36 min.

T_2_ maps of the GM and WM were reconstructed from a 3D Carr‐Purcell‐Meiboom‐Gill (CPMG) imaging sequence acquired with the following scan parameters: resolution 2.5 × 2.5 × 5 mm^3^; TR: 1250 ms; no signal averaging; and acquisition bandwidth: 20 kHz. A total of 10 echoes were acquired with a constant echo spacing of 20 ms; images were reconstructed separately for each of the 10 echo times (TEs).

Separate scans were used to more accurately map the T_1_ and T_2_ of CSF due to their much longer values than for WM/GM. For T_1_: resolution: 2.5 × 2.5 × 5 mm^3^; TR/TE/TE_eff_ = 12000 ms/11 ms/500 ms (low‐to‐high Cartesian k‐space trajectory); echo train length: 90; and inversion times: 500 ms, 1500 ms, 2000 ms, 2500 ms, 3000 ms, and 4000 ms. Total scan duration for the T_1_ mapping sequence was around 36 min. Given the relatively coarse spatial resolution, the influence of partial voluming of voxels with CSF and WM/GM could be problematic, which is why a long effective TE using low‐to‐high k‐space coverage (approximately 5 times the T_2_ of WM/GM) was used to suppress the signal from the WM/GM. T_2_ maps of the CSF were reconstructed from 7 different turbo spin echo scans: TR = 10,000 ms; echo train length = 60; and TE/TE_eff_ = 15/450, 30/900, 45/1350, 60/1800, 75/2250, 90/2700, 105/3150 ms (low to high Cartesian k‐space coverage). Total acquisition time was around 42 min.

### 2.4 Lower leg relaxation time mapping

T_1_ maps: spatial resolution: 2.5 × 2.5 × 5 mm^3^; TR/TE/TE_eff_ = 850 ms/10 ms/10 ms; echo train length: 4; no signal averaging; imaging bandwidth: 20 kHz. Scans were acquired with 6 different inversion times: 25, 50, 75, 100, 150, and 400 ms. Total scan duration for the T_1_ mapping sequence was around 36 min. T_2_ maps: spatial resolution: 2 × 2 × 6 mm^3^; TR: 800 ms; TE: 10 different TEs 11 to 110 ms in 11 ms steps; and no signal averaging. Scan duration was approximately 28 min.

### 2.5 Data processing

All images were reconstructed using the Numpy fast Fourier transform implementation, with k‐space filtered using a sine‐bell‐squared filter. Parameter maps were reconstructed from a central slice of the 3D reconstruction on a voxel‐by‐voxel basis using the least squares function in SciPy 1.6.0 running in Python 3.7.3. For T_1_ mapping the data were fitted to:

(1) $$s\left(TI,TR,\rho ,T_{1}\right)=\left|\rho \left(1‐2e^{\frac{‐TI}{T_{1}}}+e^{\frac{‐TR}{T_{1}}}\right)\right|.$$

The initial guesses for ρ and $T_{1}$ provided to the least squares function are given by:

(2) $$\rho_{\mathrm{initial}}\left(x,y\right)=\max\left(\left|s\left(x,y,TI\right)\right|\right)$$

(3) $$T_{1,initial}\left(x,y\right)=TI\left[\mathrm{argmin}\left(\left|s\left(x,y\right)\right|\right)\right]\times \ln\left(2\right).$$

For T_2_ mapping, the data were fitted to:

(4) $$s\left(TE,\rho ,T_{2}\right)=\rho e^{\frac{‐TE}{T_{2}}}.$$

The initial guesses for ρ and $T_{2}$ are given by:

(5) $$\rho_{\mathrm{initial}}\left(x,y\right)=s\left(x,y,TE\left[0\right]\right)$$

(6) $$T_{2,initial}\left(x,y\right)=TE\left[\mathrm{argmin}\left|s\left(x,y,TE\right)‐e^{‐1}s\left(x,y,TE\left[0\right]\right)\right|\right].$$

**FIGURE 1:**
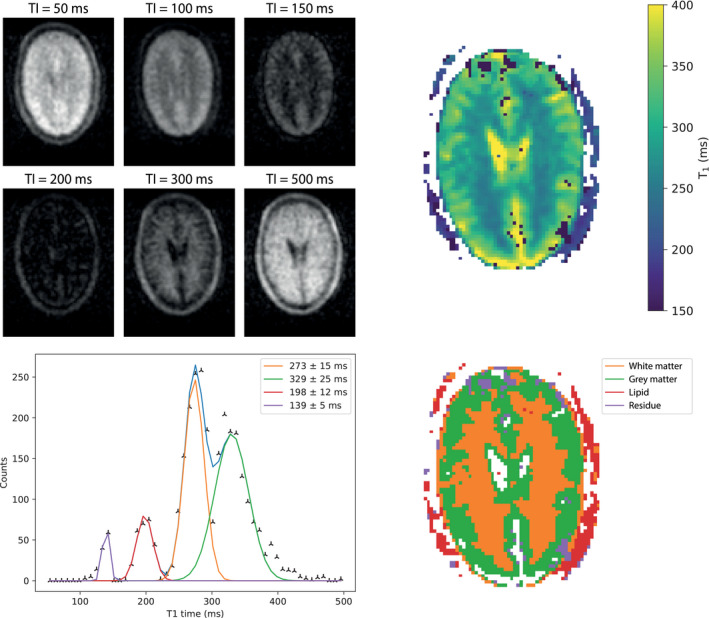
(Top left) Six brain images (central slice) acquired using an inversion‐recovery sequence with different inversion times. (Top right) A T_1_ map calculated from the acquired images. (Bottom left) A plot of binned T_1_ values; 4 Gaussian curves are fit to the histogram. (Bottom right) A segmented map of the brain with the color corresponding to the area under each fitted curve: orange is assigned to white matter, green to gray matter, red to lipid, and purple is residual.

Gaussian curves were fit to the histogram, as in Ref. [24] using the curve‐fit fitting function from SciPy 1.6.0. A segmented map was generated for each relaxation time map based on the fitted curves; data points within 2 SDs of the mean were assigned to a curve. If 2 curves overlapped, the crossing point between the 2 curves was set as the value limit for each of the tissue types (eg, WM was limited to less than 300 ms and GM to more than 300 ms in Figure 1).

## 3 RESULTS

Figure 1 shows a T_1_ map acquired from 1 of the healthy volunteers showing T_1_ contrast between GM and WM as well as lipid. It should be noted that the T_1_ values obtained in the CSF are inaccurate due to saturation effects, resulting from incomplete longitudinal relaxation due to the short repetition time (1250 ms) relative to the T_1_ time (around 4 s), which is why a separate scan was performed for accurate quantification. The measured T_1_ times for each individual are reported in Table 1: the mean T_1_ times across all 3 subjects are 327 ± 10 ms in the GM and 275 ± 5 ms in the WM.

**TABLE 1:** Measured T_1_ and T_2_ relaxation times in healthy volunteers

	GM	WM	CSF	GM (Bottomley)	WM (Bottomley)
T_1_ (ms)	329 ± 25	273 ± 15	3528 ± 192		
	335 ± 19	280 ± 14	4033 ± 325	325 ± 55	242 ± 41
	316 ± 19	271 ± 7	3546 ± 134		
T_2_ (ms)	97 ± 10		1687 ± 249		
	100 ± 8		1448 ± 226	101 ± 13	92 ± 20
	108 ± 10		1626 ± 253		

Values (where available) from Bottomley et al^22^ are calculated for a field strength of 50 mT.

Abbreviations: GM, gray matter; WM, white matter.

**FIGURE 2:**
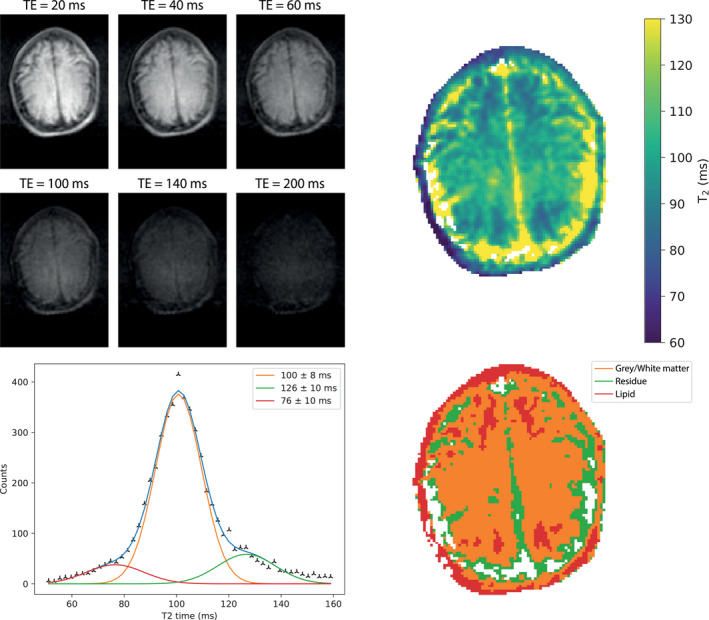
(Top left) images acquired with different TEs using a TSE sequence. (Top right) A T_2_ map reconstructed from images acquired with 10 different TEs. (Bottom left) A plot of binned T_2_ times; 3 Gaussian curves are fit to the data; the sum of the 3 curves is shown in blue. (Bottom right) A segmented map of the brain with the color corresponding to the curves of the Gaussian fit: red corresponds to lipid, orange to gray, and white matter (no distinction is possible) and green to incorrect fitting of the T_2_ of CSF. TSE, turbo spin echo

A T_2_ map of the brain of a different volunteer is shown in Figure 2. The mean T_2_ measured in both the GM and WM across the 3 subjects is 102 ± 6 ms. Note that the T_2_ of times of CSF are not accurately represented in this data set due to T_1_ saturation effects.

**FIGURE 3:**
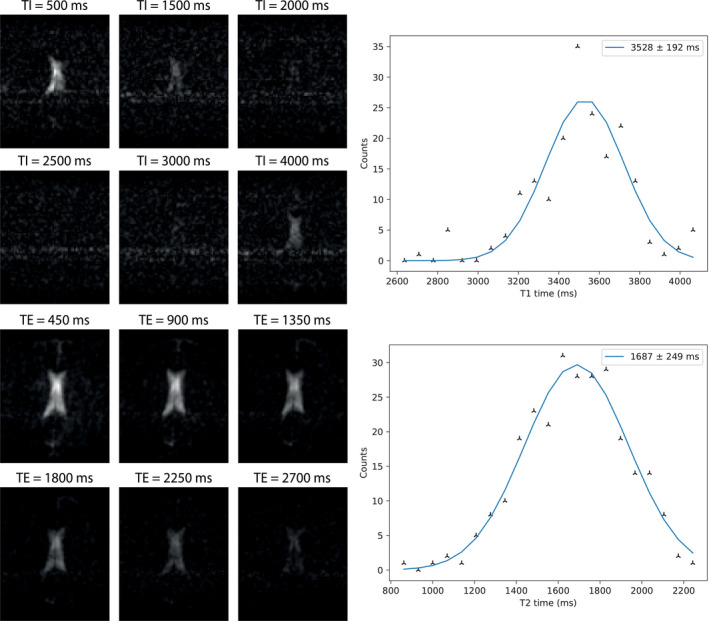
(Top left) Images acquired using an inversion recovery sequence with 6 different inversion times for CSF measurement: the background signal from WM/GM is suppressed by using a very long effective TE. (Top right) A plot of binned T_1_ times with a single Gaussian curve fitted through the data. (Bottom left) Images acquired using a TSE sequence, with 6 different TEs with (bottom right) a corresponding Gaussian fit

Figure 3 shows the measured T_1_ and T_2_ times in the CSF using a very long repetition time to allow for full longitudinal relaxation. The measured T_1_ and T_2_ times in the CSF for each subject are reported in Table 1 (note that T_1_ and T_2_ maps were not acquired in the same subjects). The mean T_1_ in the CSF is measured to be 3695 ± 287 ms, and the mean T_2_ is 1584 ± 124 ms.

**FIGURE 4:**
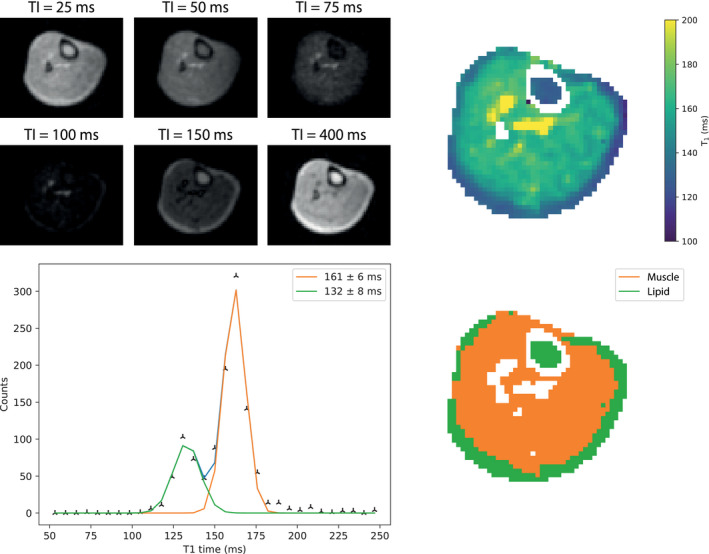
(Top left) Images acquired using an inversion recovery sequence with 6 different inversion times. (Top right) A T_1_ map reconstructed from the acquired images. (Bottom left) A corresponding histogram plot. Two Gaussian curves are fit to the histogram. (Bottom right) A segmented map of the images with the colors (orange: muscle; green: lipid) corresponding to the area under the fitted curves of the same color

For muscle and lipid measurements in the lower leg, Figure 4 shows a T_1_ map of 1 of the subjects reconstructed from 6 inversion recovery acquisitions, which are also shown. The mean T_1_ across all 3 subjects is 171 ± 11 ms in the muscle and 130 ± 5 ms in the lipid. Two areas of much higher T_1_ values are located at the anterior and posterior tibial arteries and veins and are likely due to flow effects and the relatively short repetition time relative to the expected T_1_ of blood of around 400 ms^20^: these areas are excluded from the analyses.

**FIGURE 5:**
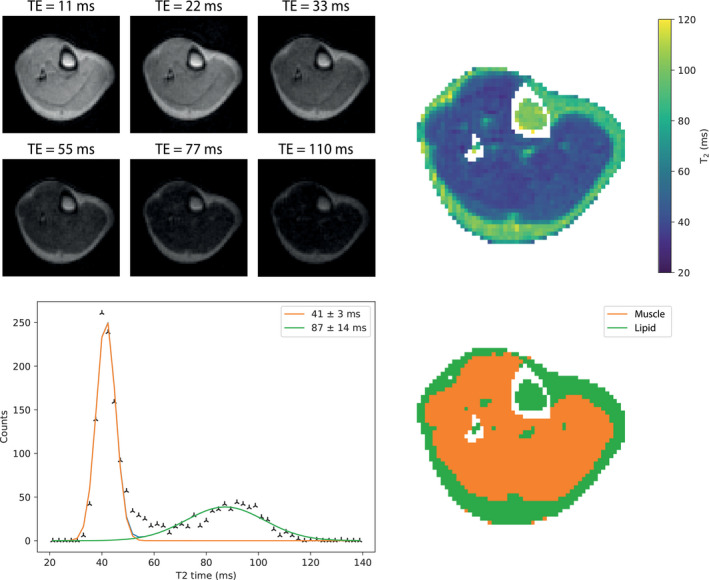
(Top left) Subset of images acquired with different TEs using a TSE readout. (Top right) A T_2_ map reconstructed from the images; images were acquired with 10 different TEs. (Bottom left) A histogram of the T_2_ map with 2 Gaussian functions fitted. (Bottom right) A segmented map of the images with the colors (orange: muscle; green: lipid) corresponding to the area under the fitted curves of the same color

Figure 5 shows a T_2_ map, together with individual images from a subset of the different TEs. The mean T_2_ across all 3 subjects for muscle is 39 ± 2 ms and 90 ± 13 ms for lipid. There is no measurable difference in the T_1_ and T_2_ between subcutaneous and bone marrow lipid. The measured T_1_ and T_2_ times in the muscle and lipid for each individual are reported in Table 2.

**TABLE 2:** Measured T_1_ and T_2_ times in the lower leg of healthy volunteers

	Muscle	Lipid	Muscle (Bottomley^22^)	Lipid (Bottomley^22^)
T_1_ (ms)	168 ± 10	134 ± 7	208 ± 37	143 ± 40
	183 ± 10	124 ± 11		
	161 ± 6	132 ± 7		
T_2_ (ms)	41 ± 3	87 ± 14	47 ± 6	84 ± 30
	40 ± 4	104 ± 10		
	37 ± 2	78 ± 14		

Values for lipid represent the combination of subcutaneous and bone marrow lipids.

## 4 DISCUSSION

In this work, we acquired in vivo T_1_ and T_2_ maps of the brain (WM, GM, CSF) and lower leg (muscle, subcutaneous lipid, bone marrow) at 50 mT. As expected, T_1_ times are lower at 50 mT than at clinical field strengths; gray and white matter T_1_s were measured to be 327 ± 10 ms and 275 ± 5 ms, respectively, compared to 1200 ms and 650 ms at 1.5 T^20^ and 1331 ms and 832 ms at 3 T.^32^ For the lower leg, T_1_ times of 171 ± 11 ms for muscle and 130 ± 5 ms for lipid were measured. Again, these are much shorter than at high field, with T_1_ of the muscle reported as 1130 ms and 1420 ms for 1.5T, and 3T and a T_1_ of 250 ms and a range 380‐450 ms for subcutaneous lipid at 1.5T^33^ and 3T,^32^ respectively. The measured T_2_ times closely match data reported at different field strengths: this is also to be expected given the relative insensitivity of T_2_ with respect to B_0_.

In terms of previous work on the field dependence of relaxation times, fundmental work on T_1_ relaxation times was performed by Koenig and Brown^34^ over a range of 0.01 to 20 MHz. Bottomley^22^ provided an extensive review of the literature and, based on this collation, proposed an empirical formula of the form:

(7) $$T_{1}=A\nu^{B}\pm SD,$$

with different values of A and B for various tissues, for example, A = 0.00362, B = 0.3082, SD = 17% (GM); A = 0.00152, B = 0.3477, SD = 17% (WM); A = 0.000455, B = 0.4203, SD = 18% (skeletal muscle); and A = 0.0113, B = 0.1743, SD = 28% (lipid). At 50 mT, these correspond to T_1_ values of ~320 ± 55 ms for GM, ~240 ± 40 ms for WM, ~210 ± 38 ms for skeletal muscle, and ~145 ± 40 ms for lipid. Rooney^20^ derived a similar formulation with T_1_ = 0.583(B_0_)^0.382^ (WM) and T_1_ = 0.857(B_0_)^0.376^ (GM). These equations correspond to values of 186 ms (WM) and 286 ms (GM). (It should be noted that experimental data with a lower limit of 0.15T were considered in this study; thus, no claim to accuracy below this field strength was made, see page 313 in^20^). Fischer et al^23^ extended the model of Bottomley to incorporate both the “physically plausible and experimentally encountered” low‐field (L) and high‐field (H) limits:

(8) $$\frac{1}{T_{1}}=H+\frac{1}{A\left(f^{B}+L\right)}=\frac{1}{T_{1,w}}+D+\frac{A}{1+{\left(\frac{f}{f_{c}}\right)}^{\beta^{\prime }}}.$$

where 1/T_1,w_ is the relaxation rate of pure water (0.23 s^−1^ at 37°C) ; D is the baseline; A is the height of the dispersion step; f_c_ is the inflection frequency; and *β*′ is the steepness of the dispersion step. Using a sample of 13 WM and 10 GM samples from 4 different brains, they empirically derive values of D (−1.52 s^−1^ WM, 0.11 s^−1^ GM), A (19.07 s^−1^ WM, 11.08 s^−1^ GM), fc (0.067 MHz WM, 0.085 MHz GM), and *β*′ (0.251 WM, 0.438 GM). This gives values of 230 ms for WM and 395 ms for GM.

Placing our experimental results in the context of these different models: T_1_(GM) of 327 ± 10 ms is right at the center of the range from Bottomley, well above the value from Rooney and below the value from Fischer; T_1_(WM) of 275 ± 5 ms is at the upper end of the range from Bottomley, well above the value from Rooney and above that from Fischer; T_1_(skeletal muscle) of 171 ± 11 ms is at the lower end of the Bottomley range; and T_1_(lipid) of 130 ± 5 ms is in the center of the Bottomley range.

Comparing to previous in vivo low field studies, Agartz et al^35^ reported brain relaxation time data at 0.02 T, with T_1_ values of 200‐220 ms for WM and 185‐200 ms for GM: these values are lower than our measured ones, in line with the lower magnetic field used. Dean et al^36^ performed in vivo relaxation time measurements of breast tissue (lipid) at 20 mT. The range of T_1_ values was 96‐133 ms, which is shorter than those we measured, as expected.

T_2_ values are generally assumed to be independent of frequency, with Bottomley reporting values of 101 ±13 ms for GM, 92 ± 20 ms for WM, 47 ± 6 ms for skeletal muscle, and 84 ± 36 ms for lipid. Our measured values in vivo agree well with these predictions. T_2_ values of 92‐98 ms for WM and 81‐87 ms for GM were also reported in the study by Agartz et al at 20 mT, which are very similar to our results. T_2_ values for lipid in breast at 20 mT were 57‐74 ms in the work by Dean, which is slightly lower than our values.

The relaxation times of CSF are notoriously difficult to measure in vivo, and a wide variety of values have been reported in the literature, in particular for the T_1_ values^20^, ^37^, ^38^, ^39^, ^40^: at 1.5T, reported values range from 3836 ± 470 ms to 4282 ms, and at 3T from 3817 ± 424 to 6873 ms. One report states that the T_1_ time for CSF shows no significant B_0_ dependence, with a value of around 4400 ms from 0.15T to 7T.^20^ Yamashiro et al^37^ showed that the T_1_ of CSF (measured at 1.5 and 3T) was significantly greater than that of pure water at room temperature. They ascribed this difference to be mainly due to the higher temperature in the body. Tsukiashi^41^ showed that the T_1_ of water increases from approximately 3.2 s at 25°C to 4 s at body temperature, whereas the T_2_ is much less temperature‐dependent, rising from 2.1 to 2.2 s. Qin^38^ mapped both the volume and T_2_ of CSF, with values at 3T of greater than 2000 ms for ventricle, but lower values (~1600 ms) for frontal cortex and ~1500 ms for both temporal cortex and occipital cortex. The paper attributed the lower T_2_ values measured in the subarachnoid spaces to be caused by a higher partial pressure of oxygen, which would correlate with the lower T_1_ values measured by Zaharchuk et al.^42^, ^43^ Other potential causes included higher protein concentration, although this was not verified or substantiated. T_2_ values of ~1400 s were measured by Spijkerman et al at 7T.^39^ Hopkins^40^ performed measurements at a variety of lower field strengths, reporting values of 4360 for the T_1_ and 1760 for the T_2_ at 0.15T. Placing our results in context, the average T_1_ value was ~3700 ms and T_2_ ~1550 ms, in line with the various literature references.

The major challenges in accurate relaxation time mapping at low field are partial volume effects resulting from the relatively coarse spatial resolution and the low intrinsic SNR. In this work, we used very conventional and somewhat time‐inefficient inversion recovery and multiple spin‐echo sequences. We were only able to measure 1 parameter per imaging session of ~45 min, with separate measurements also necessary for CSF due to its very long T_1_ and T_2_ relaxation times. In all the data, there was some evidence of multi‐exponential behavior, although we believe that the source of this is the partial volume effect rather than the tissue itself.

## 5 CONCLUSION

Practical relaxation time mapping for image segmentation purposes in longitudinal studies, or characterization of pathologies in patients, obviously will require much faster scanning. Reconstruction techniques such as compressive sensing from undersampled k‐space data have already been shown to be applicable to low‐field imaging.^44^ The application of techniques such as Look‐Locker for T_1_ ^29^ and variable tip‐angle turbo spin echo sequences that allow longer echo trains to be acquired^45^ would also be valuable to study. Finally, we note that, although strong B_0_ inhomogeneity is known to cause an underestimation of the T_2_ times due to diffusion effects,^46^ in our case typical line widths over the entire 3D imaging volume were less than 1 kHz and therefore unlikely to introduce significant errors.

The T_1_ and T_2_ times measured in this work show that generating inter‐tissue contrast based purely on relaxation times will be more challenging at low field than at higher fields. The absolute difference in T_1_ relaxation times between tissues becomes smaller at low field, which means conventional methods for generating T_1_ weighting in images, such as inversion recovery‐based sequences and imaging with a short repetition time, may be suboptimal. It may be that other contrast‐generating techniques such as magnetization transfer^47^or diffusion^48^ are required for generating strong tissue contrast at low field. Additionally, lipid suppression becomes challenging at low field because inversion recovery‐based suppression methods such as short tau inversion recovery (STIR) will also largely suppress the muscle signal due to their very similar T_1_ relaxation times (T_1,lipid_ = 130 ± 5 ms, T_1,muscle_ = 171 ± 11 ms), and the very small chemical shift of less than 10 Hz makes spectrally selective‐based approaches such as spectral presaturation with inversion recovery (SPIR) and DIXON impractical.^49^

Finally, in this preliminary work, we did not differentiate between age and gender, both of which have been shown to affect relaxation times.^50^, ^51^, ^52^ Low‐field MRI provides a low(er) cost pathway to understanding how these tissue parameters evolve over time and can be a very useful tool in longitudinal studies. T_1_ and T_2_ times are also known to correlate with disease progression^53^, ^54^, ^55^, ^56^; by making MRI scanners more accessible, there is an opportunity to scan people at a regular interval to gain a more accurate understanding of the current disease state.
